# Dasatinib and quercetin senolytic treatment delays early onset intervertebral disc degeneration in SM/J mice

**DOI:** 10.21203/rs.3.rs-6838819/v1

**Published:** 2025-06-19

**Authors:** Makarand Risbud, Emanuel Novais, Olivia Ottone, Esther Akande, Ruteja Barve

**Affiliations:** Thomas Jefferson University; Thomas Jefferson University; Thomas Jefferson University; Thomas Jefferson University; Washington University

**Keywords:** SM/J mice, senescence, intervertebral disc, degeneration, SASP, transcriptome, senolytics, dasatinib, quercetin

## Abstract

Genetic background is a major determinant of disc degeneration, a leading cause of chronic back pain and disability. Herein, we demonstrate that premature disc cell senescence contributes to early-onset degeneration in SM/J mice and test two systemic senotherapeutic strategies to mitigate it: Navitoclax (Nav.) and a cocktail of Dasatinib and Quercetin (DQ). While Nav. treatment did not improve severe degeneration in SM/J mice, DQ-treated mice showed lower grades of degeneration and decreased abundance of senescence markers p19^ARF^ and p21. DQ improved disc cell viability and phenotype retention and retarded fibrosis of the nucleus pulposus tissue. Transcriptomic analysis showed disc compartment-specific effects of the treatment, with cell cycle regulation and JNK signaling being commonly affected across tissue types. A comparison with DQ-mediated aging-dependent amelioration of disc degeneration in C57BL/6N mice identified *Junb* and *Zfp36l1* signaling as shared DQ targets in the mouse disc. This study reinforces the efficacy of senolytic treatments in ameliorating local senescence and intervertebral disc fibrosis.

## INTRODUCTION

Low back pain (LBP) and neck pain rank among the top causes of years lived with disability^1^. Though the etiology of LBP is multifactorial, patients with intervertebral disc degeneration are three times more susceptible to LBP^2^. The intervertebral disc sandwiched between the adjacent vertebrae confers spinal flexibility and accommodates loading^3^. This ability results from the interaction of the disc compartments: the central, glycosaminoglycan-rich nucleus pulposus (NP); the circumferential, annulus fibrous (AF), comprised of highly organized collagen fibrils; and endplates (EP), which consist of a thin layer of hyaline cartilage and a subchondral bone plate^4^. Each compartment is distinguished by its extracellular matrix, which maintains largely non-proliferative cells adapted to accommodate the physiological avascular and hypoxic conditions of the disc^5,6^. Degeneration affects each disc compartment, and abnormal function of any of them influences the degenerative cascade of the others^7^. Broadly, the degenerative process is characterized by altered extracellular matrix (ECM) organization and composition^8^, loss of biomechanical properties^9^, increased inflammatory mediators and catabolic processes, changes in cell phenotype, cell death^10^, and senescence^11,12^.

Among many factors contributing to disc degeneration, genetic predisposition is one of the major contributors to the disease process^13^. Battié et al. demonstrated that genetics is the top predisposing factor to disc degeneration, followed by aging and loading in humans^14^. Many studies have described a correlation between the disease and several single-nucleotide polymorphisms related to extracellular matrix^15^, matrix catabolism^16^, inflammation^17^, and cell signaling^18^. More recently, we and others have shown that the genetic background governs the susceptibility to disc degeneration and the progression into specific disease sub-phenotypes, including fibrosis, ectopic calcification and herniation in mice^19–21^.

Mechanistic studies of disc degeneration have been hampered by the need for appropriate animal models recapitulating human pathology without genetic manipulation or injury. Recent studies have shown that the SM/J, an inbred mouse strain, which exhibits poor healing ability, first described in the context of cartilage regeneration, undergoes spontaneous disc degeneration, replicating key molecular, phenotypic, and functional features, including aging-associated disc herniation and back pain in humans^9,22,23,^. However, the cellular mechanisms driving early-onset disc degeneration in SM/J mice remain relatively unexplored.

Different studies have shown the contribution of senescence to intervertebral degeneration in humans and mice^10,11^. Senescence is broadly characterized by cell cycle arrest, apoptotic resistance, and the production of inflammatory and catabolic factors known as the senescence-associated secretory phenotype (SASP)^24^. The increased expression of cell cycle inhibitors such as p21, p53, p16^INK4a^, and p19^ARF^ across tissues are hallmarks of this cell stage^35^. This senescent cell state causes local fibrosis, loss of regenerative capacity, and, ultimately, tissue degeneration^24^. Genetic and natural aging models have shown that targeting senescence modulates the progression of disc disease and back pain^10,25,26^. Similarly, using cultured human disc cells, Cherif *et al*. showed effective clearing of senescent disc cells reduced inflammatory signaling following senolytic intervention^27^; however, there is limited knowledge about the applicability and success of senolytic treatments to target different phenotypes of disc degeneration in vivo.

Senolytic therapies, which selectively induce apoptosis of senescent cells, have gained substantial traction in musculoskeletal pathologies since they were first described in 2015^48^. Several compounds, such as ABT-263 (Navitoclax, Nav.), which targets the BCL-2 pathway^28^; BCL-XL inhibitors like A1331852 and A1155463^29^; flavonoids, including Quercetin, Fisetin, and Piperlongumine; and Src/tyrosine kinase inhibitors^30^ are shown to successfully remove senescent cells. Among the senotherapeutic compounds being studied, the combination of Dasatinib (D) – a Src/tyrosine kinase inhibitor – and Quercetin (Q) – a natural flavonoid that binds to BCL-2 and modulates transcription factors, cell cycle proteins, pro- and anti-apoptotic proteins, growth factors, and protein kinases^31^ – (DQ) has shown the most promising results, with low toxicity^32^. Accordingly, DQ was the first senolytic approach used in clinical trials, showing efficiency in clearing senescent cells in humans and improving and promoting physical function^33,34^. In the context of disc degeneration, we have recently shown that systemic treatment with DQ cocktail can effectively reduce the age-associated senescence burden and disc degeneration in C57BL/6N (B6N) mice^35^.

In this study, we demonstrate that early-onset, spontaneous disc degeneration in SM/J mice is associated with elevated senescence burden. Importantly, we determined the efficacy of systemic DQ and Nav. treatment in targeting senescence in the disc and alleviating the early onset degenerative process. Notably, our results show that DQ, but not Nav., reduces the severity of disc degeneration and senescence burden through targeting *Jun* and *Zfp36l1* signaling pathways. This work further supports the potential of systemically delivered DQ to ameliorate the effects of early onset, spontaneous disc degeneration, and contribute to deciphering the mechanisms of senotherapeutic systems, which may support future clinical applications.

## RESULTS

### SM/J mice show a high senescence burden which coincides with the progression of disc degeneration

**Figure 1:**
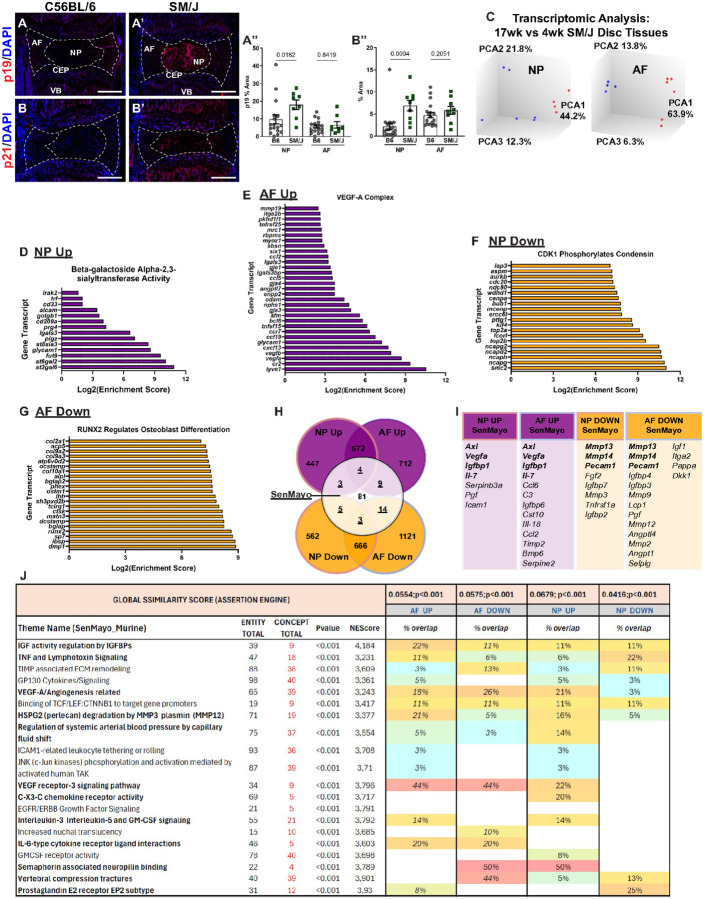
Caudal discs of SM/J mice evidence early cellular senescence and a senescence signature during degeneration. At four weeks of age, SM/J mice have increase abundance of senescence markers in their caudal discs, evidenced by **(A-A”)** p19 and **(B-B”)** p21 abundance relative to age-matched B6J discs. **(C)** Microarray analysis of 4-week-old and 17-week-old SM/J NP and AF shows distinct clustering in both tissues between the two timepoints. Thematic analysis in CompBio of enriched concepts in 17-week-old NP tissues, compared to 4-week-old tissues shows: **(D’)** *Beta-galactoside Alpha-2,3-sialytransferase Activity* is an upregulated theme in the NP; **(E)** *VEGF-A Complex* is an upregulated theme in the AF; **(F)** *CDK1 Phosphorylates Condensin* is a downregulated theme in the NP; and **(G)** *RUNX2 Regulates Osteoblast Differentiation* is a downregulated theme in the AF. **(H)** Venn Diagram showing the gene-level overlap between SM/J tissue profiles and the *SenMayo* geneset, with the overlapping genes shown in **(I)**. **(J)** Global similarity score (assertion engine) results, including all overlapped themes, concepts and DEGs between SM/J tissues (4–17wk) and the *SenMayo* gene set. Data are shown as mean ± SD. Significance was determined using an unpaired t-test or Mann-Whitney test, as appropriate. n_C57BL/6J 4w_ = 8; n_SM/J 4w_ = 5; n_SM/J 17w_ = 6.

SM/J mice show early onset, spontaneous disc degeneration, recapitulating several salient features of human degeneration by the time animals are 17 weeks old^9,22^. Considering the role of cellular senescence in intervertebral disc degeneration^10,11,32^, we investigated the senescence status of SM/J discs at 4 weeks, prior to the conspicuous cell death in the NP compartment^9^. Interestingly, 4-week-old SM/J caudal discs presented higher levels of senescence markers p19 (Fig. 1A–A”) and p21 (Fig. 1B–B”), compared to C57BL/6J (B6J) mice, which expresses these markers with aging, between 18–24 months^10,32^. To investigate the contribution of cell senescence to progression of degeneration in SM/J mice, global transcriptomic analysis was conducted on 4- and 17-week-old NP and AF tissues, which showed distinct profiles at the both timepoints (Fig. 1C). To gain insight into the functional implications of these transcriptomic changes, the CompBio analysis tool (PercayAI Inc., St. Louis, MO) was used to determine thematic associations among differentially expressed genes^36,37^ (full results in Suppl. Table 1). Notably, analysis of upregulated DEGs in 17-week-old SM/J NP tissue showed enrichment for *Beta-galactoside Alpha-2,3-sialytransferase Activity* and *EPH-Ephrin Signaling*, which have implications in cellular senescence and response to senolytic compounds, such as Dasatinib^38,39^ (Fig. 1D, Fig. S1A). In the AF transcriptome, signatures associated with *VEGF-A Complex, IL1, and Megakaryocytes in Obesity, Hypokalemic Alkalosis*, and *Negative Regulation of TORC2 Signaling* increased during degeneration, demonstrating several molecular hallmarks of disc degeneration (Fig. 1E, Fig. S1. B-B”). In line with these findings, downregulated themes in the NP enriched around several matrix and cell cycle-related themes, including *CDK1 phosphorylates condensing and Transcription of E2F Targets, Heparan Sulfate 2-O-sulfotransferase Activity, and TNFR1-induced NFkB signaling pathway* (Fig. 1F, Fig. S1C-C”). Similarly, the downregulated themes in the AF were enriched for processes such as *RUNX2 regulates osteoblast differentiation, Arp2/3 Complex Binding, and Sos-mediated nucleotide exchange of Ras* (Fig. 1G, Fig. S1D-D’).

To more precisely investigate the correlation between the disc degeneration process in SM/J model with established senescence signatures, concept-level assertion engine analysis was conducted on CompBio outputs for up- and downregulated concepts in NP and AF tissues, compared to the published SenMayo gene set^40^, revealing significant associations in both tissues. Cross-referencing the DEG gene lists from NP and AF against the SenMayo dataset revealed several shared genes (Fig. 1H). Specifically, *Axl, Vegfa, Igfbp1*, and *Il7* were shared between NP/AF up-regulated DEGs and senescence, whereas *Mmp13, Mmp14*, and *Pecam1* were common to NP/AF down-regulated DEGs and senescence (Fig. 1I). At the thematic level, among the top 20 themes identified from SenMayo biological enrichment, all but one - “EGFR/ERBB Growth Factor Signaling” - overlapped with the degenerative signaling observed in the SM/J 17-week intervertebral disc (Fig. 1J). Themes that showed a high degree of overlap, included “IGF Activity Regulation by IGFBPs,” “TNF and Lymphotoxin Signaling,” “HSPG2 (Perlecan) Degradation by MMP3/Plasmin (MMP12),” “C-X3-C Chemokine Receptor Activity,” “IL-6-Type Cytokine Receptor-Ligand Interactions” “Vertebral Compression Fractures,” and “Prostaglandin E2 Receptor EP2 Subtype” (Fig. 1J).

These findings suggest that cellular senescence contributes to the degeneration observed in SM/J mice, and therefore, we sought to intervene in this degenerative progression using senotherapeutics.

### DQ treatment, but not Navitoclax, improves degenerative and senescence outcomes in SM/J mice

**Figure 2:**
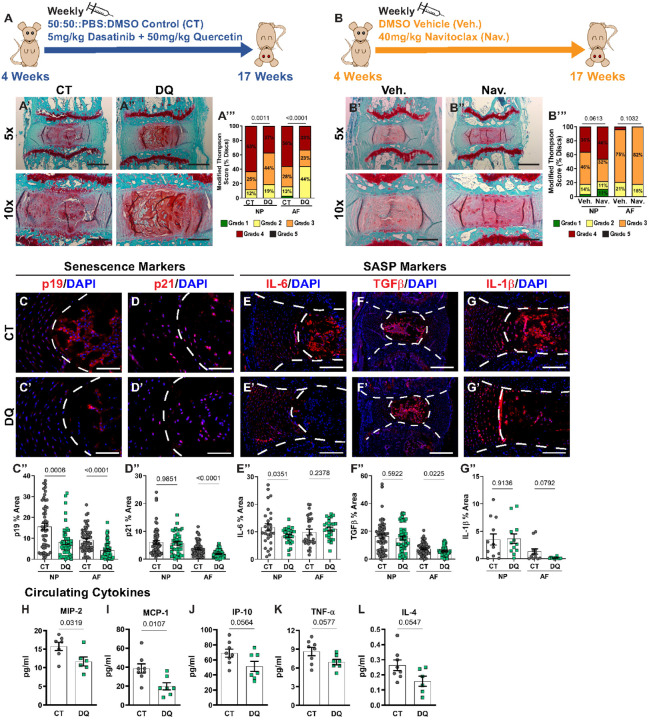
DQ reduces caudal disc degeneration and senescence in SM/J mice. **(A, B)** Schematic showing study design: intraperitoneal injections of DQ, Nav., or a Vehicle control were administered once every week to mice starting at 4 weeks of age and ending at 17 weeks of age. **(A’-A’”)** SafraninO/Fast Green/Hematoxylin staining evaluated with modified Thompson scoring shows DQ improves disc degeneration in SM/J mice. Images reflect the range of degenerative outcomes across treatment cohorts. **(B’-B”)** Safranin/Fast Green/Hematoxylin staining evaluated with modified Thompson scoring shows Nav. does not improve disc degeneration in SM/J mice. Quantitative immunohistochemistry shows reduced **(C-C”)** p19 (NP and AF) and **(D-D”)** p21 (AF only) in DQ-treated SM/J discs. SASP markers of **(E-E”)** TGF b, **(F-F”)** IL-6, and **(G-G”)** IL-1b indicate DQ mediates SASP in SM/J discs. Plasma analyses show lower levels of **(H)** MIP-2 and **(I)** MCP-1 in DQ-treated SM/J mice, and downward trends were observed in **(J)** IP-10, **(K)** TNF-a, and **(L)** IL-4. Data are shown as mean ± SD. Significance was determined using an unpaired t-test or Mann-Whitney test, as appropriate. Distribution statistics were determined using a χ^2^ test. 17 weeks old (n_DQ_ = 26, 13 females + 13 males; n_CT_ = 20, 12 females + 8 males; n_Nav._ = 7, n_Veh._ = 7).

Previously reported successful outcomes of DQ treatment in aging B6N mice were dependent on the age when the treatment was initiated, showing the maximum efficiency when administered during the early stages of the disease process, suggesting a finite window for local cellular response and plasticity^35^. Accordingly, beginning at 4 weeks of age until 17 weeks, SM/J mice received either a weekly treatment with Dasatinib (5 mg/kg) (D) and Quercetin (50 mg/kg) (Q) combination (DQ) (Fig. 2A) or Navitoclax (Nav.) (40 mg/kg) (Fig. 2B) to target senescent cells and ameliorate disc degeneration. Histological analysis of discs showed better tissue preservation, cellularity, and cell morphology, with better NP/AF compartment demarcation and fewer AF clefts relative to vehicle-treated control animals (CT) (Fig. 2A’–A”). Improvements to the disc architecture were observed in the DQ treatment cohort as early as 6–8 weeks (Fig. S2A-B”). Further, modified Thompson grading showed a reduction of approximately 25% in severely degenerated (grade 4) NP and AF tissues (Fig. 2A’”)^19,41^. By contrast, Navitoclax-treated mice did not demonstrate structural improvements in their discs, evidenced by histological analysis and modified Thompson scoring (Fig. 2B–B’”). Accordingly, discs of the DQ-treated cohort were further evaluated to understand how DQ reduced disc degeneration in SM/J mice.

To better understand the cellular processes underlying the structural improvements to the disc, several indicators of cell senescence and SASP were evaluated at the tissue level, and the plasma cytokine profile was determined^29,30,45–47^. In both NP and AF tissues of DQ-treated mice, p19^ARF^ (p19) levels were reduced (Fig. 2C–C”), and p21 abundance was reduced in the AF (Fig. 2D–D”). These observations provided evidence of a reduced senescence burden in the disc tissues of SM/J mice by DQ treatment. Complementary analysis of SASP markers showed reduced IL-6 (Fig. 2E–E”) in the NP, reduced TGFb (Fig. 2F–F”), without affecting IL-1b levels (Fig. 2G–G”) in the AF of DQ-treated mice. These changes indicated a possible reduction in local inflammation and pro-fibrotic signaling with DQ treatment and suggested that DQ effectively reduces the incidence and severity of disc degeneration in SM/J mice by mitigating cell senescence and SASP^42,43,58–60^.

To study the effect of DQ treatment on systemic cytokine levels, we measured several pro-inflammatory molecules in plasma. Notably, DQ mice showed decreased levels of proinflammatory proteins MIP-2 (Fig. 2H) and MCP-1 (Fig. 2I), with trends toward reduction in IP-10 (Fig. 2J) (*p* = 0.0564), TNF-a (Fig. 2K) (*p* = 0.0577), and IL-4 (Fig. 2L) (*p* = 0.0547). This response was selective as we noted a lack of change in several other plasma cytokines in DQ-treated mice (Fig. S3). These results showed that DQ treatment mitigated tissue-level pro-inflammatory response and attenuated systemic inflammation in SM/J mice.

To further evaluate the systemic impact of DQ treatment on SM/J mice, the caudal vertebral bone was analyzed using micro-computed tomography (μCT). Three-dimensional reconstructions of the caudal vertebrae (Fig. S4A, A’) showed no changes in the vertebral length (Fig. S4B), disc height (Fig. S4C), or disc height index (Fig. S4D). In the trabecular bone, the bone volume fraction (BV/TV) (Fig. S4E), trabecular thickness (Tb. Th.) (Fig. S4F), and trabecular number (Tb. N.) (Fig. S4G) were unchanged, while the DQ-treated cohort evidenced a slight reduction in trabecular spacing (Tb. Sp.) (Fig. S4H). This change is unlikely to bear functional significance due to its small magnitude and the absence of change in other parameters. Evaluation of the cortical bone (Fig. S4I, I’) showed DQ did not impact bone volume (BV) (Fig. S4J), area (B. Ar.) (Fig. S4K), perimeter (B. Pm.) (Fig. S4L), or cross-sectional thickness (Cs. Th) (Fig. S4M). Together, these results show that DQ treatment minimally affects the vertebral bone, suggesting its safe systemic use for other musculoskeletal tissues.

### DQ treatment attenuates degeneration by limiting NP tissue fibrosis.

**Figure 3:**
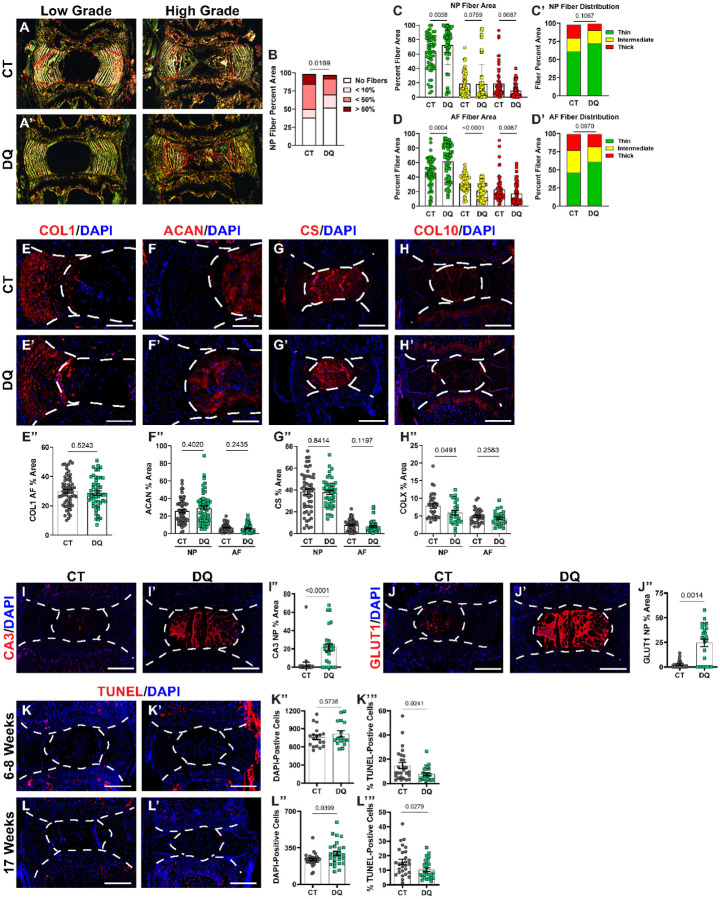
DQ-treated discs show reduced NP fibrosis, NP cell phenotype retention, and improved cell survival. **(A-A’)** Quantitative picrosirius red staining indicates that **(B**) DQ-treated discs evidence less NP fibrosis, **(C-C’)** with higher proportion of thin (green) collagen fibers in DQ-treated NP tissues, and **(D-D”)** AF collagen fiber thickness being significantly altered by DQ treatment. Quantitative immunohistological staining shows no change in **(E-E”)** COL1, **(F-F”)** ACAN, or **(G-G”)** chondroitin sulfate (CS) abundance in DQ-treated mice. **(H-H”)** COL10 abundance was significantly reduced in the NP by DQ treatment. Significantly improved retention of NP phenotypic markers **(I-I”)** CA3 and **(J-J”)** GLUT1 is observed in DQ mice. **(K-K’”)** At 6–8 weeks, there are not changes in cellularity in DQ mice, but there is a reduction in TUNEL-positive cells. **(L-L’)** By 17 weeks, TUNEL staining shows **(L”)** improved cellularity and **(L’”)** a reduction in apoptosis in the discs of DQ-treated mice. Data are shown as mean ± SD. Significance was determined using an unpaired t-test or Mann-Whitney test, as appropriate. Distribution statistics were determined using a χ^2^ test. n_DQ_ = 5–7, n_CT_ = 5–7, 3–4 levels each animal.

ECM is essential for proper disc function. In SM/J mice, degeneration culminates in the fibrotic remodeling of the matrix, marked by a decrease in proteoglycans and increased collagen deposition^20,57^, resulting in NP fibrosis, and consequent loss of mechanical properties^9,23^. Major structural proteins in the disc were evaluated specifically to study fibrotic remodeling in DQ-treated discs. Aligning well with the Modified Thompson Scores of DQ-treated discs, analysis of picrosirius red staining (Fig. 3A–A’) showed approximately 25% fewer discs in the DQ cohort had collagen fibers in the NP compartment (Fig. 3B); healthy discs do not have appreciable collagen deposition in the NP. When the fibrotic NP tissues were analyzed, there were no quantitative differences in the collagen fiber thickness in tissues from the DQ and CT cohorts (Fig. 3C–C’). Analysis of collagen fiber thickness in the AF showed that DQ mice had thinner collagen fibers than CT (Fig. 3D–D’), suggesting DQ delays the fibrotic degenerative phenotype of SM/J mice by increasing collagen remodeling. Interestingly, the abundance of Collagen I (COLI) (Fig. 3E–E’), the aggrecan core protein (ACAN) (Fig. 3F–F”), and chondroitin sulfate (CS) staining (Fig. 3G–G”) were similar between the vehicle and DQ treated cohorts, suggesting that the structural collapse of the disc during degeneration precedes increased degradation of these matrix proteins at 17 weeks. On the other hand, DQ treatment led to the reduction of collagen 10 (COL10), often associated with the acquisition of a hypertrophic chondrocyte-like phenotype by NP cells suggesting that DQ facilitates the retention of the NP cell phenotype.

### DQ treatment preserves NP cell phenotype.

Since NP cells in SM/J mice are known to progressively differentiate into chondrocyte-like cells, we investigated DQ’s effects on NP cell phenotype and viability^61^. Carbonic anhydrase 3 (CA3) and glucose transporter 1 (GLUT1) are known NP phenotypic markers whose abundance decreases during disc degeneration and aging^23,37^. Accordingly, NP cells from DQ-treated mice robustly expressed CA3 (Fig. 3I–I”) and GLUT1 (Fig. 3J–J”), and the CT group showed a decreased abundance of these markers. Similarly, discs of DQ-treated mice showed higher NP cellularity and lower percentages of TUNEL-positive cells as early as 6–8 weeks of age (Fig. 3K–K”’), resulting in retention of a higher number of cells and consistently lower TUNEL-positive cells at 17-weeks (Fig. 3L–L’”). This suggests that DQ treatment mitigates senescence in the disc by preserving the NP cell phenotype and improving cell viability.

### DQ treatment results in a distinct transcriptomic signature in the AF and NP compartments.

**Figure 4:**
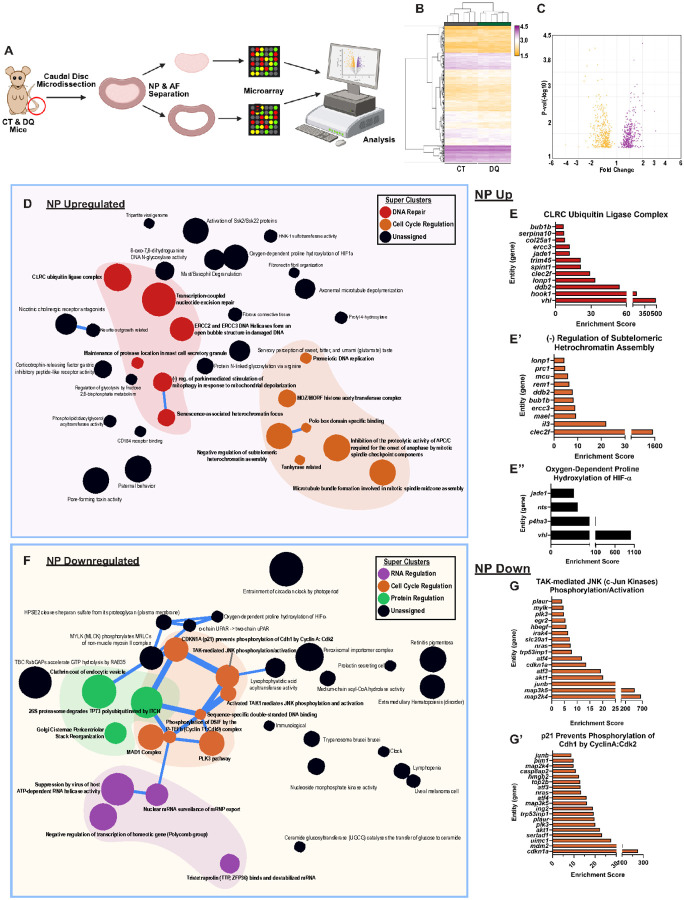
Transcriptomic analysis of NP tissues highlights possible mechanisms by which DQ improves SM/J disc degeneration. **(A)** Schematic showing the microarray workflow for analyzing DQ-treated NP and AF tissues. **(B-C)** Hierarchical clustering of microarray data and volcano plots of 859 DEGs identified in the NP demonstrate distinct clustering of CT and DQ cohorts in NP tissues. **(D-E”)** Compbio analysis highlighted themes relating to DNA repair (red) and cell cycle (orange) among upregulated DEGs in the NP and **(F-G’)** themes relating to the cell cycle (orange), RNA regulation (purple), and protein regulation (green) among the downregulated DEGs in the NP. 4-week-old (n = 6), 17-week-old CT and DQ mice (n = 6 mice/treatment).

**Figure 5:**
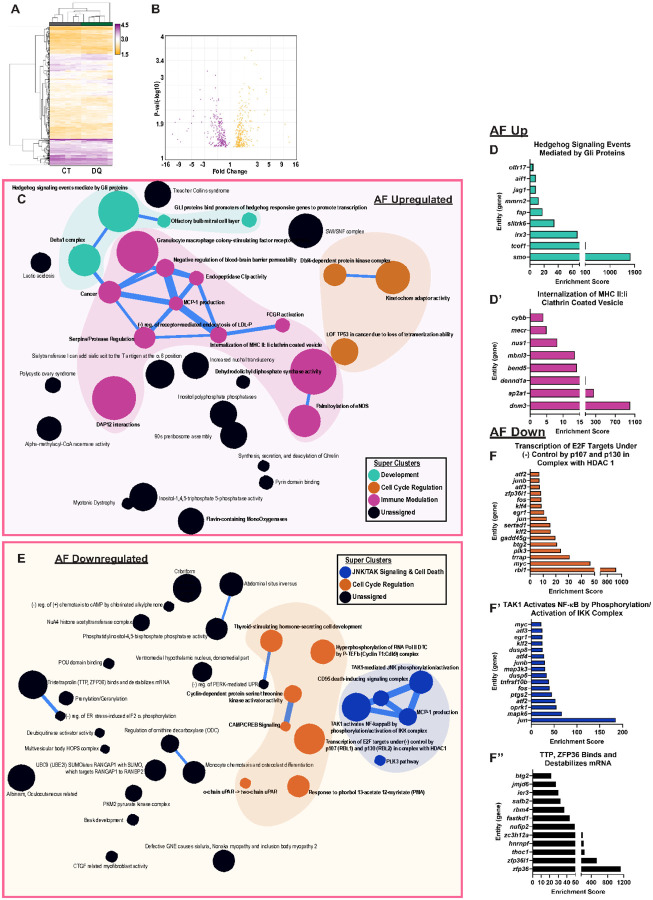
Transcriptomic analysis of AF tissues highlights possible mechanisms by which DQ improves SM/J disc degeneration. **(A)** Hierarchical clustering of microarray data demonstrates distinct clustering of CT and DQ cohorts in AF tissues. **(B)**Volcano plot showing 323 upregulated and 263 downregulated DEGs in the AF of DQ-treated mice. **(C-D’)** Compbio analysis of AF tissues highlighted themes relating to development (turquoise), cell cycle (orange), and inflammatory signaling (pink) among upregulated DEGs and **(E-F”)** themes relating to the cell cycle (orange) and JNK/TAK signaling/cell death (blue) among the downregulated DEGs. 17-week-old CT and DQ mice (n = 6 mice/treatment).

To better understand the possible mechanisms underlying the observed phenotypic improvements in SM/J mice receiving DQ, we performed a global transcriptomic analysis of the NP and AF tissues from 17-week-old CT and DQ cohorts (Fig. 4A). We assessed the baseline differences between treatment groups by analyzing the differentially expressed genes (DEGs, defined by p ≤ 0.05) in NP (Fig. 4B, C) and AF (Fig. 5A, B) tissues. Hierarchical clustering analysis demonstrated distinct transcriptomic profiles for CT and DQ groups in both tissues (Fig. 4B, Fig. 5A). We identified 382 upregulated DEGs and 441 downregulated DEGs in the NP; 311 upregulated DEGs, and 242 downregulated DEGs in the AF; and 12 commonly upregulated and 21 commonly downregulated DEGs between compartments (Fig. S5A). Commonly upregulated DEGs included *Sel1l2, Lonp1, Tmem160, Raly*, and *Mgat2*; and common downregulated DEGs included *Atf3, Ier2, Zfp36l1, Junb*, and *Plaur* (Fig. S5B).

To better understand the biological impact of the DEGs, the CompBio tool (PercayAI Inc., St. Louis, MO) was used to conduct pathway-level analysis (Fig. 4D–G’, Suppl. File 1’). In the NP tissues of DQ-treated mice, several related themes forming thematic clusters relating to DNA repair (red cluster) and cell cycle regulation (orange cluster) were identified (Fig. 4D), along with notable themes including *CLRC Ubiquitin Ligase Complex, Negative Regulation of Subtelomeric Heterochromatin Assembly, and Oxygen-Dependent Proline Hydroxylation of HIF-a* (Fig. 4E–E”). Among the downregulated genes, there was also a significant signature relating to cell cycle regulation (orange cluster), which appeared to coalesce around themes relating to CDKN1A and JNK/TAK signaling (Fig. 4F’), along with significantly enriched themes for *TAK-mediated JNK Phosphorylation/Activation and p21 Prevents Phosphorylation by Cdh1 by CyclinA:Cdk2* (Fig. 5G–G’). Additionally, there were several themes relating to RNA (purple cluster) and protein (green cluster) regulation (Fig. 4F). Notably, both up- and downregulated DEGs showed themes relating to proline hydroxylation of HIF, and among the downregulated DEGs, there were themes relating to the circadian clock and the cleavage of heparan sulfate from its core proteoglycan.

In the AF, hierarchical clustering also revealed distinct clustering between control and DQ-treated groups (Fig. 5A–B). AF upregulated DEG analysis presented several themes related to development (turquoise cluster), cell cycle (orange cycle), and immune modulation (pink cluster) (Fig. 5C). Notable themes within these clusters included *Hedgehog Signaling Events Mediated by Gli Proteins and Internalization of MHC II: Ii Clathrin Coated Vesicle* (Fig. 5D–D’). Among the downregulated themes, there was again a substantial cell cycle signature (orange cluster), and interestingly, there was a cluster of themes specifically related to JNK/TAK signaling and cell death (Fig. 5E). This is highlighted in themes of *Transcription of E2F Targets Under Negative Control of p107 and p130 in Complex with HDAC1; TAK1 Activates NF-kB by Phosphorylation/Activation of OKK Complex; and TTP, ZFP36 Binds and Destabilizes mRNA and* supports the previous observations that suggest DQ improves degenerative outcomes through the negative regulation of cell cycle arrest and apoptosis (Fig. 5F–F’).

**Figure 6:**
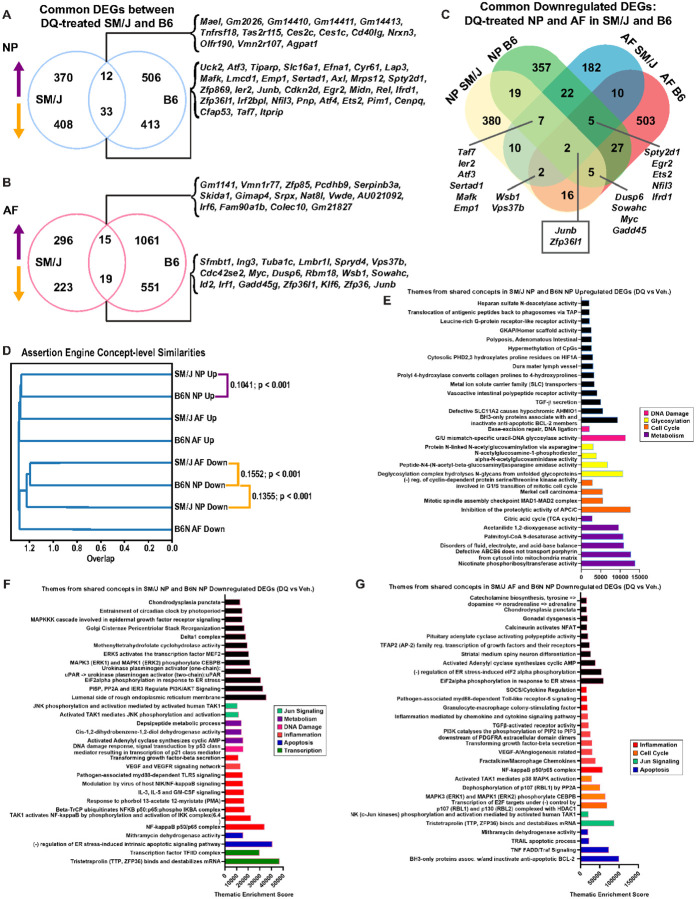
Comparative analysis of transcriptomic data from DQ-treated SM/J mice and aged B6N mice reveal pathways commonly regulated by DQ. **(A)** Comparison of DEGs in the NP of DQ-treated SM/J and B6N mice shows 12 commonly upregulated DEGs and 33 commonly downregulated DEGs. **(B)** Comparison of DEGs in the AF of DQ-treated SM/J and B6N mice shows 15 commonly upregulated DEGs and 19 commonly downregulated DEGs. **(C)** Analysis of downregulated DEGs in NP and AF tissues from SM/J and B6N mice reveals *Junb* and *Zfp36l1* are the only two commonly downregulated DEGs **(D)** Assertion engine analysis identifies three comparisons to be the most similar at the concept level across treatment cohorts and tissues: upregulated by DQ in SM/J NP and B6N NP; downregulated by DQ in SM/J NP and B6N NP; and downregulated by DQ in SMJ AF and B6N NP. **(E)** Significant themes upregulated by DQ in SM/J NP and B6N NP **(F)** significant themes downregulated by DQ in SM/J NP and B6N NP **(G)** significant themes downregulated by DQ in SMJ AF and B6N NP.

Beyond understanding the impact of DQ in SM/J discs, the understanding of the molecular mechanisms by which DQ ameliorates the degeneration process in the intervertebral disc is limited. To gain further mechanistic insights, we compared the transcriptomic data from DQ-treated SM/J mice to previously reported findings from DQ-treated aged B6N mice. Direct comparison of the DEGs in the NP from these two mouse models identified 12 upregulated and 33 downregulated common DEGs (Fig. 6A). In the AF, 15 upregulated and 19 downregulated DEGs were common to DQ treatment in SM/J and B6N mice (Fig. 6B). When the downregulated DEGs were compared across both mouse models and disc tissues (NP and AF), we found that only two transcripts were commonly downregulated: *Junb* and *Zfp36l1*, important regulators of senescence fate (Fig. 6C). Moreover, these cross models and disc tissues’ common transcripts fortify previous NP and AF gene signature analysis, suggesting JUN signaling as a critical convergence point conferring the benefits of the systemic DQ treatment on disc health.

Though the common downregulation of *Junb* and *Zfp36l1* is a substantial lead into how DQ may mediate disc degeneration, two genes/pathways are insufficient to fully capture the processes driving improved disc health outcomes. Accordingly, we then analyzed the concepts generated in CompBio from DQ vs. CT DEG comparisons to understand the biological processes common to the two treatment models at the pathway level. Assertion engine analysis identified three comparisons to be the most similar at the concept level: upregulated by DQ in SM/J NP and B6N NP; downregulated by DQ in SM/J NP and B6N NP; and downregulated by DQ in SMJ AF and B6N NP (Fig. 6D). Themes that emerged from the SM/J and B6N NP upregulated comparison related to DNA damage, glycosylation, cell cycle, and metabolism; and the SM/J and B6N NP downregulated comparisons had signatures for Jun signaling, metabolism, DNA damage, inflammation, apoptosis, and transcription (Fig. 6E). The comparison between SM/J AF and B6N NP downregulated concepts overlapped with many of these, including inflammation, cell cycle, Jun signaling, and apoptosis (Fig. 6F, G). Notably, these results suggest that in the context of both aging and genetic predisposition models of disc degeneration, DQ improves health outcomes by reducing cell death, and suppressing the activation of inflammatory pathways and that *Junb* may be central to this process.

## DISCUSSION

**Figure 7:**
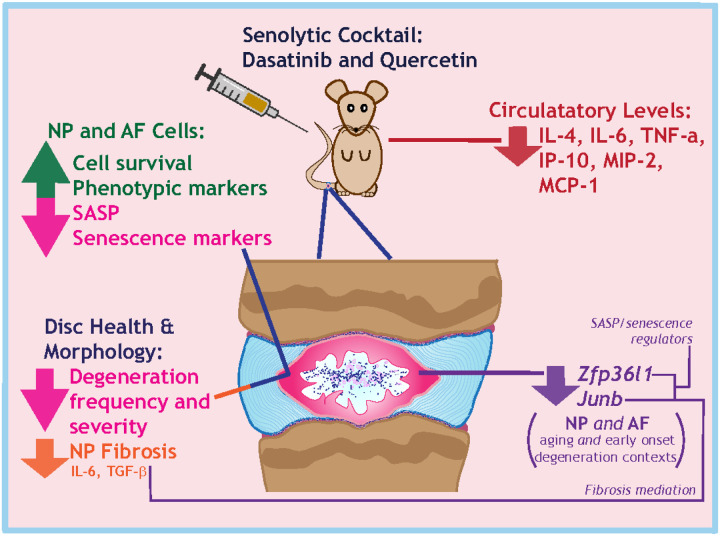
Schematic summarizing the improved degenerative outcomes of SM/J discs treated with DQ. SM/J mice treated with DQ from 4 weeks of age until 17 weeks of age evidence reduced frequency and severity of intervertebral disc degeneration, marked by reduction in degenerative scores and reductions in fibrosis and fibrotic markers. This was associated with improved disc cell survival, a retention of disc phenotypic markers, and a reduction in senescence and SASP markers. DQ treatment also alleviated systemic inflammation in SM/J mice. Transcriptomic analysis revealed *Zfp36l* and *Junb* as potential regulators of these processes, as they are known to be upstream of master senescence regulators and involved in the regulation of IL-6 and TGF-b.

Despite the high global incidence and associated costs of intervertebral disc degeneration and chronic back and neck pain, clinical interventions remain primarily limited to symptomatic relief and non-disease modulation^43^. This clinical reality is, in part, a result of the complexities underlying disc degeneration and its multifactorial etiology. Among the processes contributing to disc degeneration, cellular senescence is prevalent in degenerative tissues, and its mitigation has shown promise in delaying disc degeneration and back pain^10,26,32^. Due to its positive correlation with age, senescence is often studied within the context of aging or, in progeria models, posing practical challenges to understanding its contribution to a wide gamut of disc pathologies^11,25,42^. The recently described SM/J mouse, a model of early-onset, spontaneous disc degeneration, offers an avenue to study disease phenotypes without using strategies of genetic manipulations or injury to expedite the disease process^9,22^. Notably, SM/J mice have a comparable lifespan to other inbred strains, such as C57BL/6 and LG/J^23^. Herein, we demonstrate a high senescence burden characterized by p19 and p21 abundance in SM/J discs as early as 4 weeks of age, and the NP and AF transcriptomic profiles during the 17-week degeneration process capture features in the established SenMayo gene set, suggesting that cell senescence is part of their degenerative process^40^. This follows previous work suggesting that senescence in the disc is not solely linked to aging but more broadly to degeneration^11^. After establishing a correlation between tissue-level senescence and disc degeneration, we investigated the potential of two senotherapeutics – Navitoclax (Nav.) and a Dasatinib and Quercetin (DQ) combination – to ameliorate disc degeneration in SM/J mice, which showed promising outcomes for the DQ cocktail. By cross-referencing the transcriptomic signature of DQ SM/J mice with our previous work on aging B6N mice^23^, we found that DQ reduces degenerative outcomes by limiting cell death, and the downregulation of *Junb* and *Zfp35l1* as key players in this process (Fig. 7). Importantly, this work establishes SM/J mice as a model to study senescence in disc degeneration and contributes to evidence of senotherapeutics working by preventing disease progression rather than the classical mechanism of selective killing of senescent cells to promote tissue repopulation.

In human intervertebral discs, a positive correlation between degeneration and local senescence is established^11^. Additionally, in aging mice, systemic elimination of cells positive for p16^INK4a^, an important marker of cell senescence^43^, demonstrates a clear causality between disc degeneration and senescence^25^. In recent years, senotherapeutics have been shown to selectively target senescent cells in a variety of cell and tissue types by interfering with their unique pro-survival pathways, such as JAK1/2, BCL-2/BCL-XL, PI3K/AKT, p53/p21/Serpines, dependence receptors/tyrosine kinases, and the HIF-1α pro-survival mechanism^44^. Among these therapeutics, Navitoclax (ABT263) has shown promise in chondrocytes, cartilage tissue culture, and hip explant cultures, with results demonstrating the ability of the drug to selectively clear senescent cells and reduce SASP^45^. Similarly, in an injury-induced model of disc degeneration, local injection of Nav. to the injured disc improved structural degeneration and reduced the local senescence burden and SASP^46^. By contrast, our results demonstrate that systemic Nav. administration is insufficient to reduce degenerative outcomes in SM/J mice. This finding suggests that the efficacy of Nav. in the disc is limited to the context of local administration or possibly dependent on the local concentration of the drug. Intradiscal injection, however, poses the risk of propagating damage to the disc by introducing a new acute injury, as suggested by animal studies and a landmark study on discography in human patients by Carragee and colleagues, necessitating further investigation of potential mitigators of disc degeneration that can be systemically delivered^47^. Additionally, our results suggest that, in the context of disc senescence, simultaneous inhibition of ephrin B (using Dasatinib) and the PI3K/AKT pathways (using Quercetin) is more effective than targeting BCL-XL/BCL-W and MCL-1 with Nav^48^. These findings are in line with the recent reports by Sanborn et al., which underscore the tissue-specific nature of senescence signatures and further emphasize that the efficacy of senotherapeutic strategies is highly context-dependent, varying according to tissue type, therapeutic window, and dosage^12,26,49^.

One of the major consequences of tissue degeneration in a vast majority of age-related diseases, such as dementia, glaucoma, chronic obstructive disease, and musculoskeletal pathologies, are local fibrosis and loss of matrix homeostasis^50^. The intervertebral disc is no exception, with fibrosis being one of the major disc degeneration subphenotypes characterized by reduced shock absorption, spine flexibility, and disc height, culminating in back pain^9,20^. We have previously shown that p16, a master regulator of senescence, modulates SASP and matrix composition during aging in the intervertebral disc^42^. Similarly to the aging B6N model, DQ treatment in the present study promoted lower rates of NP fibrosis, evidenced by lower TGF-β levels^51^. Moreover, DQ promoted retention of the NP cell phenotype, with the lower acquisition of a hypertrophic chondrocyte-like phenotype, demonstrated by the reduced abundance of COLX^52^. While DQ treatment did not achieve total mitigation of disc degeneration, it may have improved the local plasticity of cells to respond to stressors, delaying degeneration and promoting local extracellular matrix function. In this context, modulation of Arp2/3 signaling and actin cytoskeleton by DQ treatment supports this rationale by implying cellular osmoadaptation to the local environment^53^.

Systemic DQ treatment has been shown to effectively target senescent cells^9^ in human disease contexts, with a growing number of clinical studies investigating its efficacy for disorders ranging from fibrotic NAFLD to skeletal health during aging^33,34^. Previously, DQ showed positive effects in the context of age-associated disc degeneration^35^. In the current study, we tested the DQ regimen in the SM/J mice, a model of early-onset disc degeneration, one of the main causes of back pain in middle-aged adults^54^. As was previously observed in B6N mice treated with DQ, complete rescue of the degenerative phenotype was not observed in SM/J mice; however, significant improvements to tissue and cellular morphology were observed at 6–8 weeks and 17 weeks. Improved morphological outcomes were accompanied by a reduction in senescence markers and SASP in NP and AF tissues, indicating that systemic DQ treatment can successfully modulate cell behavior in the disc microenvironment. Of particular interest are our findings on reduced cell death and better retention of NP cell phenotypic markers in the discs of DQ-treated SM/J mice. This was also observed in the DQ aging B6N mice model, where DQ treatment improved degenerative outcomes by limiting cell senescence, which prevents SASP, cell death, and consequent degeneration^35^. Senescent cells are typically considered to have entered a state of permanent cell cycle arrest, and the central dogma of senolytic drugs is that they selectively kill senescent cells, enabling non-senescent cells to repopulate the tissue with healthier cells and better maintain tissue homeostasis^44^. Our results, however, suggest that DQ impacts the disc in an alternative or complementary fashion, promoting cell survival and retention of the native cell phenotype, which limits cell death and degeneration of the disc. The cells in the disc are post-mitotic, so if senolytic drugs led to the death of senescent cells, it is unlikely the remaining cells would repopulate the compartment, which is shown by a lower rate of cell loss between 6–8 and 17 weeks in DQ-treated SM/J mice. Additionally, initiating cell death in a sparsely populated compartment could potentially further propagate damage to the tissue^55^. Supporting this idea, there is evidence that cell senescence could be reversible through p16/p53 axis^56,57^ providing some basis for the hypothesis that DQ senotherapeutics enabled the survival of the existing cells and preserved their phenotype. Our findings are mimicked by senostatics, which, for example, in osteoarthritis, can modulate STING and NF-kB pathways, preventing apoptosis and senescent cell fate^58,59^. While in the context of the disc, the cGAS-STING pathway alone does not prevent senescence progression^60^, prolonged activation of NF-kB has been shown to accelerate disc degeneration through increased local production of inflammatory cytokines, chemotactic proteins, and catabolic enzymes^61^. Thus, we hypothesize that DQ inhibition of JUN signaling, and consequently JUN-NF-κB cross-talk, may play a crucial role in promoting disc cell survival and maintaining disc tissue homeostasis^62^. These findings support the idea that the success of a senotherapeutic regimen is dependent on tissue type, pathology, administration route/time, and the signaling pathways it may targets^35,63^.

Notably, when overlapping the *universal organismal aging genes* in mice, comprising 76 cell-type-specific signatures from Tabula Muris Senis, with established senescence marker panels, Jun emerged as one of the only three common upregulated genes^49^. Consistently, both DQ-treated SM/J mice and aged B6N models showed downregulation of JNK pathway activation, known to regulate senescence and *Cdkn2a* (*p16*) expression, accompanied by reduced senescence markers across intervertebral disc compartments^64^. Similarly, a shared downregulation across NP and AF tissues of *Junb* and *Zfp36l1* was noted in these models. It is important to note that JUNB has been shown to regulate the feedforward network of TGFb signaling promoting sustained activation of genes involved in cell adhesion, ECM function, and epithelial-mesenchymal transition^65^. Similarly, JUN forms a positive regulatory circuit with an important SASP factor IL6, thereby promoting a profibrotic response^66^. These findings suggest that the downregulation of *Junb* by DQ treatment not only attenuates senescence-associated features, including cell cycle arrest and SASP markers, but may also directly contribute to reduced fibrosis, decreasing the expression of critical local and systemic mediators, such as IL-6 and TGF-β. This positions JUNB as a potential therapeutic target for the alleviation of disc senescence and intervention in disc fibrosis.

Regarding disc cell homeostasis, in both models, at a thematic level, DQ treatment resulted in downregulating various genes related to DNA damage, inflammation, and apoptosis. Reductions in DNA damage and inflammatory signatures may be indicative of lower senescence and SASP burdens in the tissues, and a reduced apoptotic signature further supports DQ treatment, prolonging the survival of cells. Notably, in SM/J mice, AF tissues from DQ-treated mice showed enrichment for hedgehog signaling, which is critical for maintaining disc health and could indicate one means by which DQ treatment preserves disc cell phenotypes^67,68^.

In summary, our findings in a model of early-onset disc degeneration build on previous findings in aging B6N mice and provide further evidence for the beneficial effects of systemic administration of DQ, but not Navitoclax to improve health outcomes in the disc^9,35^. We also show that SM/J mice are a model of senescence-associated disc degeneration, providing the field with the benefit of a model of spontaneous disc degeneration and senescence without the constraints of waiting for animals to age or performing complex genetic manipulations. Evidence supports that DQ treatment in both SM/J and aging B6N mice improves degenerative outcomes in the disc by promoting cell survival, limiting the progression of senescence and SASP, and ameliorating intervertebral disc fibrosis. Excitingly, our results suggest a new link between DQ treatment and JUN pathway downregulation, which may underscore the beneficial effects of DQ in NP and AF tissues, paving way for future studies to investigate this mechanism.

## MATERIALS AND METHODS

### Mice, treatment, and study design

Animal procedures were performed under approved protocols by the IACUC of Thomas Jefferson University. SM/J (Stock #000687, Jackson Labs) and C57BL/6J (Stock #000664, Jackson Labs) were bred and raised at Thomas Jefferson University. For preliminary histological analyses SM/J (n = 5) and C57BL/6J (n = 8) were collected at 4 weeks of age. Beginning at 4 weeks of age, mice received a weekly intraperitoneal injection of either 40 mg/kg Navitoclax (Nav.), 5 mg/kg Dasatinib with 50 mg/kg Quercetin (DQ), or a PBS and DMSO vehicle control (CT). Animals received this treatment until they were 6–8 weeks old (n = 7 mice/treatment group, DQ and CT only) or 17 weeks old (n_CT_ = 19 (6F, 7M), n_DQ_ = 20 (6F, 5M); n_Nav_. = 7 (3F, 4M), n_Veh_. = 7 (3F, 4M)). These timepoints were selected based on previous studies showing mildly degenerative caudal disc tissue at 4 weeks old, significant cell death at 8 weeks, and severe fibrotic disc degeneration by 17 weeks^9,22^.

### Tissue Processing, μCT Analysis, and Histology

Caudal spine motion segments Ca5-Ca9 were dissected and immediately fixed in 4% PFA in PBS at 4°C for 48 hours. Following fixation, μCT scans (Bruker Skyscan 1275; Bruker, Kontich, Belgium) were performed. An aluminum filter was used, and all scans were conducted at 50 kV and 200 μA, with an exposure time of 85 ms, yielding a resolution of 15 mm. Three-dimensional image reconstructions were generated, and all subsequent analyses were conducted using Bruker programs NRecon, CTan, and CTVox.). n_CT_=8 mice (5F, 3M), n_DQ_=6 mice (3F, 3M); 3–5 vertebrae/mouse, 4 discs/mouse.

Motion segments then underwent 21 days of decalcification in 20% EDTA at 4°C, followed by paraffin embedding. Coronal sections of 7 μm were generated, and Histoclear deparaffinization followed by graded ethanol rehydration preceded all staining protocols.

Safranin O/Fast Green/Hematoxylin staining was conducted and visualized using 5x/0.15 N-Achroplan and 20x/0,5 EC Plan-Neofluar (Carl Zeiss) objectives on an AxioImager 2 microscope and Zen2^™^ software (Carl Zeiss Microscopy). This staining was used to evaluate disc structure, and four blinded graders scored NP and AF compartments using Modified Thompson Grading. Picrosirius red staining was conducted and imaged in the brightfield and under polarized light using 4x Pol/WD 7.0 objectives on an Eclipse LV100 POL microscope (Nikon). NIS Elements Viewer software (Nikon) was then used to evaluate the areas of the disc occupied by green (thin fibers), yellow (intermediate fibers), or red (thick pixels) pixels. NP fibrosis was also quantified according the percentage of the NP space occupied by collagen fibers.

### Immunohistology and cell number measurements

For all immunohistochemical stains, antibody-specific antigen retrieval was conducted by way of incubation in either chondroitinase ABC for 30 minutes at 37°C, hot citrate solution (pH 6) for 40 minutes, or proteinase K for 8 minutes at room temperature. Tissue sections were then blocked in 2–10% normal serum in PBS-T, and incubated with antibodies against p19 (1:100, Novus NB200–106), p21 (1:200, Novus NB100–1941), collagen I (1:100, Abcam ab34710), aggrecan (1:50; Millipore; AB1031), chondroitin sulfate (1:300, Abcam ab11570), IL-1b (1:100, Novus NB600–633), IL-6 (1:50, Novus NB600– 1131), TGFb (1:100; Abcam; ab92486), collagen X (1:500, Abcam ab58632), CA3 (1:150, Santa Cruz), and GLUT-1 (1:200, Abcam, ab40084). For GLUT1, a M.O.M. kit (Vector laboratories, BMK-2202) was used for blocking and primary antibody incubation. Tissue sections were washed with PBS-T and incubated in the dark with the appropriate Alexa Fluor^®^ −594 or −488 conjugated secondary antibody (1:700; Jackson ImmunoResearch Laboratories, Inc.) for one at room temperature. TUNEL staining was conducted using an in situ cell death detection kit (Roche Diagnostic; 12156792910) according to manufacturer’s specifications. All stained sections were washed with PBS-T and mounted with ProLong(^™^) Diamond Antifade Mountant with DAPI (Fisher Scientific, P36971). Stains were visualized with an AxioImager 2 (Carl Zeiss Microscopy), using 5x/0.15 N-Achroplan and 20x/0,5 EC Plan-Neofluar objectives, an X-Cite^®^ 120Q Excitation Light Source (Excelitas Technologies), AxioCam MRm camera (Carl Zeiss Microscopy), and Zen2TM software (Carl Zeiss Microscopy). Exposure settings remained constant across treatments for each stain.

### Digital Image Analysis

All immunohistochemical quantification was conducted in greyscale using the Fiji package of ImageJ^69^. Images with a selected ROI (NP and AF EP) were thresholded to subtract the background, transformed into binary format, and then staining area and cell number were calculated using the analyze particle function in Image J software, v1.53e^9,38^.

### Circulating Cytokine Analysis

Blood was collected by intracardiac puncture following sacrifice and centrifuged at 1500 rcf, at 4°C for 15 min to isolate the plasma, which was stored at −80°C until analysis. Levels of proinflammatory proteins and cytokines were analyzed using V-PLEX Mouse Cytokine 19-Plex Kit (Meso Scale Diagnostics, K15255D) according to manufacturer’s specifications.

### Tissue RNA Isolation and Microarray Analysis

NP and AF tissues were dissected from caudal discs (Ca1-Ca15) of 4-week-old (n = 6), 17-week-old CT and DQ mice (n = 6 mice/treatment). Pooled tissue from a single animal served as an individual sample. Samples were homogenized, and total RNA was extracted using the RNeasy^®^ Mini kit (Qiagen). The purified, DNA-free RNA was converted to cDNA using the EcoDry^™^ Premix (Clontech). Template cDNA and gene-specific primers (IDT, IN) were added to Power SYBR Green master mix, and expression was quantified using the Step One Plus Real-time PCR System (Applied Biosystems).

Total RNA with RIN > 4 was used for the analysis. Fragmented biotin-labeled cDNA was synthesized using the GeneChip WT Plus kit according to the ABI protocol (Thermo Fisher). Gene chips (Mouse Clariom S) were hybridized with biotin-labeled cDNA. Arrays were washed and stained with GeneChip hybridization wash & stain kit and scanned on an Affymetrix Gene Chip Scanner 3000 7G, using the Command Console Software. Quality Control of the experiment was performed in the Expression Console Software v 1.4.1. .CHP files were generated by sst-rma normalization from Affymetrix .CEL files, using the Expression Console Software. Only protein-coding genes were included in the analyses. Detection above background higher than 50% was used for Significance Analysis of Microarrays (SAM), and the p-value was set at 5%. Gene-level analyses and visualizations were conducted in the Affymetrix Transcriptome Analysis Console (TAC) 4.0 software. Array data are deposited in the GEO database, GSE281300.

### Bioinformatic Analysis

Significantly differentially up- and downregulated genes from the NP and AF compartments were cleaned for only preotein-coding genes using PANTHER classification system database^70^ and enriched analyzed using the GTAC-CompBio Analysis Tool (PercayAI Inc., St. Louis, MO)^5,37^. CompBio performs a literature analysis to identify relevant biological processes and pathways represented by the input differentially expressed entities, in this case, DEGs. This is accomplished with an automated Biological Knowledge Generation Engine (BKGE) that extracts all abstracts from PubMed that reference entities of interest (or their synonyms), using contextual language processing and a biological language dictionary that is not restricted to fixed pathway and ontology knowledge bases. Conditional probability analysis is utilized to compute the statistical enrichment of biological concepts (processes/pathways) over those that occur by random sampling. Related concepts built from the list of differentially expressed entities are further clustered into higher-level themes (e.g., biological pathways/processes, cell types, and structures, etc.). Within CompBio, scoring of entity (DEG), concept, and overall theme enrichment is accomplished using a multi-component function referred to as the Normalized Enrichment Score (NES). The first component utilizes an empirical p-value derived from several thousand random entity lists of comparable size to the user’s input entity list to define the rarity of a given entity-concept event. The second component, effectively representing the fold enrichment, is based on the ratio of the concept enrichment score to the mean of that concept’ s enrichment score across the set of randomized entity data. As such, the NES reflects the rarity of the concept event associated with an entity list, as well as its degree of overall enrichment. Complete thematic, entity, and concept-level data for analyses conducted in control and DQ-treated NP and AF tissues are included in Supplementary File 1.

The program was further used to compare the NP and AF profiles from DQ-treated SM/J mice with deposited NP and AF profiles from DQ-treated aged B6N mice (GSE154619) at the concept level. This was done by identifying the biological terms/concepts common across datasets and running those concepts as entities to acquire common themes across projects. An assertion engine tool was also used to determine which comparisons across projects were most similar.

### Statistical analyses

All statistical analyses were performed using Prism10 (GraphPad, La Jolla). Data are represented as mean ± SD. Data distribution was assessed with the Shapiro-Wilk normality test, and the differences between the two groups were analyzed by t-test or Mann-Whitney, as appropriate. A χ^2^ test was used to analyze the differences between the distribution of percentages. p ≤ 0.05 was considered a statistically significant difference.

## Supplementary Material

Supplementary Files

This is a list of supplementary files associated with this preprint. Click to download.

- SupplFile1.xlsx
- SupplFile2.xlsx
- SenoSMJFiguresSupp.pdf

## Data Availability

The microarray dataset that supports the findings of this study is openly available in the GEO database, accession number GSE281300.
